# Expression of Concern: Knockdown of TMEM14A expression by RNAi inhibits the proliferation and invasion of human ovarian cancer cells

**DOI:** 10.1042/BSR-2015-0258_EOC

**Published:** 2023-02-09

**Keywords:** cell cycle, metastasis, ovarian cancer, THEM14A

The Editorial Office has been made aware of potential issues surrounding the scientific validity of this paper, hence has issued an expression of concern to notify readers whilst the Editorial Office investigates. Multiple images seem to appear in other manuscripts by unrelated authors:

- The Figure 5B Smad2 bands, which seem to appear as the Figure 5A STAT3 bands from Liu et al. 2016 (https://doi.org/10.1038/srep33676)
- The Figure 4C PCNA bands, which seem to appear as the Figure 4B B-catenin bands from Zhu et al. 2016 (https://doi.org/10.1038/srep19070); and the Figure 4C MMP-2 bands, which seem to appear as the Figure 4B B-catenin bands from the same paper by Zhu et al. (2016)
- The Figure 4C MMP-2 bands, which seem to appear as the Figure 2C HDAC1 bands from Wang et al. 2017 (doi: 10.18632/oncotarget.18227)
- The Figure 4C MMP-9 bands, which seem to appear as the Figure 6D VCAM1 bands (vertically flipped) from Cheng et al. 2015 (https://doi.org/10.1186/s13046-015-0193-y)
- The Figure 4D Cyclin E bands, which seem to appear as the Figure 4F CCL3 bands from Wang et al. 2015 (doi: 10.3892/or.2015.4112)

